# The connections of neocortical pyramidal cells can implement the learning of new categories, attractor memory, and top–down recall and attention

**DOI:** 10.1007/s00429-021-02347-z

**Published:** 2021-08-04

**Authors:** Edmund T. Rolls

**Affiliations:** 1 Oxford Centre for Computational Neuroscience, Oxford, UK; 2 Department of Computer Science, University of Warwick, Coventry, CV4 7AL UK

**Keywords:** Neocortex, Categorisation, Short-term memory, Top–down attention, Memory recall, Pyramidal cells, Learning

## Abstract

Neocortical pyramidal cells have three key classes of excitatory input: forward inputs from the previous cortical area (or thalamus); recurrent collateral synapses from nearby pyramidal cells; and backprojection inputs from the following cortical area. The neocortex performs three major types of computation: (1) unsupervised learning of new categories, by allocating neurons to respond to combinations of inputs from the preceding cortical stage, which can be performed using competitive learning; (2) short-term memory, which can be performed by an attractor network using the recurrent collaterals; and (3) recall of what has been learned by top–down backprojections from the following cortical area. There is only one type of excitatory neuron involved, pyramidal cells, with these three types of input. It is proposed, and tested by simulations of a neuronal network model, that pyramidal cells can implement all three types of learning simultaneously, and can subsequently usefully categorise the forward inputs; keep them active in short-term memory; and later recall the representations using the backprojection input. This provides a new approach to understanding how one type of excitatory neuron in the neocortex can implement these three major types of computation, and provides a conceptual advance in understanding how the cerebral neocortex may work.

**Supplementary Information:**

The online version contains supplementary material available at 10.1007/s00429-021-02347-z.

## Introduction

How the cerebral cortex performs its functions is a key question in neuroscience. A small region of neocortex can perform several crucial functions, namely learning new categories to represent the inputs; short-term memory; top–down recall of information; and top–down attention. The neuroanatomy of the neocortex suggests that it has one main type of principal excitatory neuron, pyramidal cells (supplemented by granule cells in layer 4). In this paper, the question that is asked is whether the known microconnectivity of the neocortex can perform all of these functions with just one main type of excitatory neuron, pyramidal cells.

A number of detailed models and summaries of neocortical microarchitecture have been described (Douglas et al. 2004; Douglas and Martin 2004; Douglas and Martin 2010; Markram 2010; Harris and Shepherd 2015; Markram and al. 2015; Gal et al. 2017; Reimann et al. 2017; Shepherd and Rowe 2017). Most anatomical studies hardly mention the computational implications of the recurrent collateral connections between local pyramidal cells, though there are some exceptions (Rolls and Treves 1998; Shu et al. 2003; Douglas and Martin 2007; Mante et al. 2013; Miller 2016; Rolls 2016a, 2021b; Kar et al. 2019). The excitatory recurrent collaterals are, it is argued, the distinguishing feature of neocortical architecture, which allow for attractor networks for short-term memory and thereby planning a sequence of actions that have to be held in short-term memory; episodic and semantic long-term memory; and decision-making, as analysed elsewhere (Rolls 2016a, 2021b). Many of the detailed anatomical descriptions of the neocortex do not consider what computational functions may be being performed by the anatomy that is described.

One key aim of this paper is to propose a theory of how all of these functions that are prototypical of the neocortex, learning new categories, short-term memory, and top–down memory recall plus top–down attention, can be performed by the single excitatory neuron type, the pyramidal cell, that is prototypical of the neocortex. (Another prototypical function of the human neocortex is language, and it has been proposed that a trajectory through a state space of attractors in different neocortical attractor networks representing different parts of speech could be used for language functions (Rolls and Deco 2015a; Rolls 2021b), and the operation of these networks is likely to involve in addition categorisation and recall). A second key aim is to provide a neural network model and simulations of it that enable the proposals to be clearly specified, and that enable the theory to be tested by investigating whether all three types of computation can self-organise in the same network, and if so, what parameters for the operation may be important, to guide future research. The model that is simulated is kept simple, to enable the key computations proposed here to be illustrated, demonstrated, and tested. For example, a dynamical simulation involving the realistic dynamics of neurons and synapses and the almost random firing times of neurons is useful for understanding decision-making (Wang 2002; Rolls and Deco 2010; Deco et al. 2013; Rolls 2021b) but is not needed for, and indeed would make over-complicated, the testing of the hypotheses presented here. Similarly, the investigation of how different neural networks perhaps in different brain areas interact is an interesting issue (Renart et al. 1999, 2001; Rolls et al. 2012; Rolls 2021b), but again would unnecessarily over-complicate the testing of the hypotheses presented here. The purpose is to propose the concepts of operation of this system in the cerebral neocortex, with simple but quantitative demonstration of the principles of operation using a Matlab program which is supplied with this paper to ensure that the system is specified exactly (CompetitiveAttractorBPNetDemo.m). The aim is thus to introduce new concepts about how the cerebral cortex may operate to perform these three key computations, categorisation, short-term memory, and memory recall plus top–down attention, with one set of neocortical pyramidal cells, with 3 classes of input. Full-scale simulations of a cortical area are left for future work. However, the types of network described here use only local synaptic modification rules, and should scale up well, with quantitative studies performed on the storage capacity of attractor (Hopfield 1982; Amit 1989; Treves and Rolls 1991) and pattern association (Rolls and Treves 1990) networks (Rolls 2016a, 2021b).

A third key aim of the present research is to provide a theory for how some key components of neocortical architecture may operate to perform three fundamentally different types of computation with a single type of principal neuron, the neocortical pyramidal cell, to complement and guide the enormous effort being invested in describing the microarchitecture of the neocortex (Rolls 2016a, 2021b).

## Methods

### The connectional microarchitecture of the neocortex

**Fig. 1:**
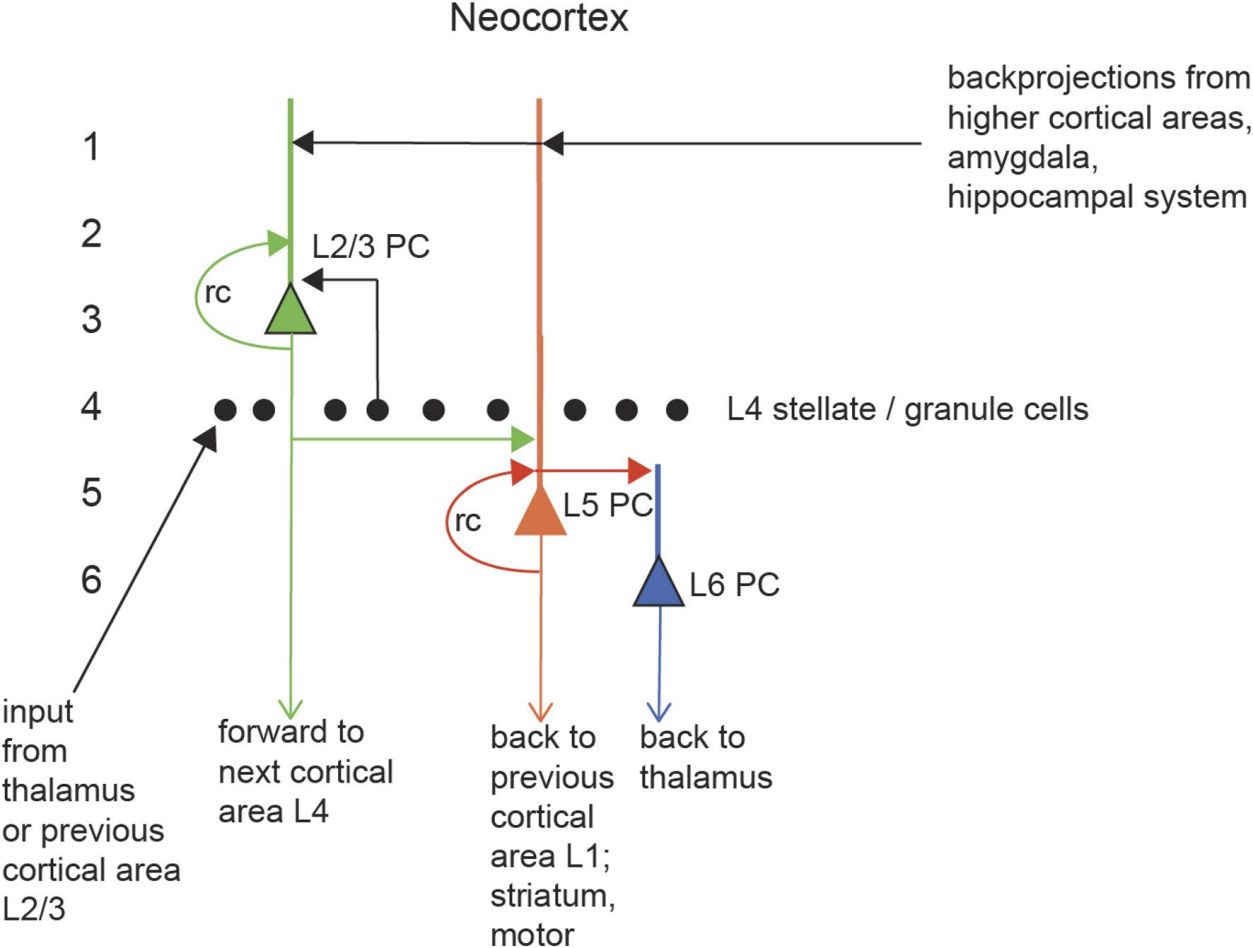
Functional canonical microcircuit of the neocortex. The cortical layers are numbered 1–6, with layer 1 at the surface of the cortex, and layer 6 adjacent to the white matter consisting of axonal connections to other brain areas. Recurrent collateral connections (rc) are shown as a loop back to a particular population of cells, of which just one neuron is shown in this Figure. In primates, the feedforward projection neurons are concentrated in L3B; and the main feedback projection neurons are in L6 and Lower L5 (L5B), but some L2 and L3A neurons do send backprojections (Markov et al. 2014). Some L6 cortico-thalamic neurons send a projection to L4 (see text). From Rolls ET (2021b) Brain Computations: What and How. Oxford University Press: Oxford

A connectional diagram of the neocortex that does include the recurrent collaterals of the pyramidal cells is shown in Fig. 1 (Rolls 2016a, 2021b). For the purposes of this paper, we will consider a population of L2/3 pyramidal cells of the type shown in Fig. 1. (The deep pyramidal cells have analogous connectivity, but different outputs (Rolls 2016a).) The architecture in the cortex is that each pyramidal cell is likely to have in the order of 10,000 recurrent collateral connections with the local population of pyramidal cells; that each neuron receives forward inputs from the previous cortical area with several thousand connections onto each neuron; and that thousands of backprojections from higher cortical areas in the hierarchy end in layer 1 on the apical dendrites of the pyramidal cells. What is shown in Fig. 1 is based on the anatomical studies cited above (Douglas et al. 2004; Douglas and Martin 2004; Douglas and Martin 2010; Markram 2010; Harris and Shepherd 2015; Markram and al. 2015; Gal et al. 2017; Reimann et al. 2017; Shepherd and Rowe 2017), and on many more described elsewhere (Rolls 2016a, 2021b). The architecture shown in Fig. 1 is qualitatively similar for different neocortical regions, with the differences between areas quantitative rather than qualitative. For example, the dendrites of pyramidal cells are generally larger and the number of synapses larger as one proceeds up a cortical hierarchy from early cortical areas to higher cortical areas (Elston and Rosa 1998; Elston 2007), but the connection principles shown in Fig. 1 are common to different cortical areas (Rolls 2016a, 2021b). The theory and simulation results described in this paper thus apply to all neocortical areas. Inhibitory neurons are not shown in Fig. 1 because they have many fewer synapses, do not connect between different neocortical areas, and accordingly are believed to be involved in housekeeping computations using feedback and feedforward inhibition to maintain the stability of the excitatory pyramidal cell populations and the sparseness of the representations implemented by the pyramidal cells, as set out elsewhere (Rolls 2016a, 2021b).

### The microarchitecture of neocortical pyramidal cells: computational theory

#### Proposal: the forward inputs act as a competitive network to implement unsupervised learning of new categories

**Fig. 2:**
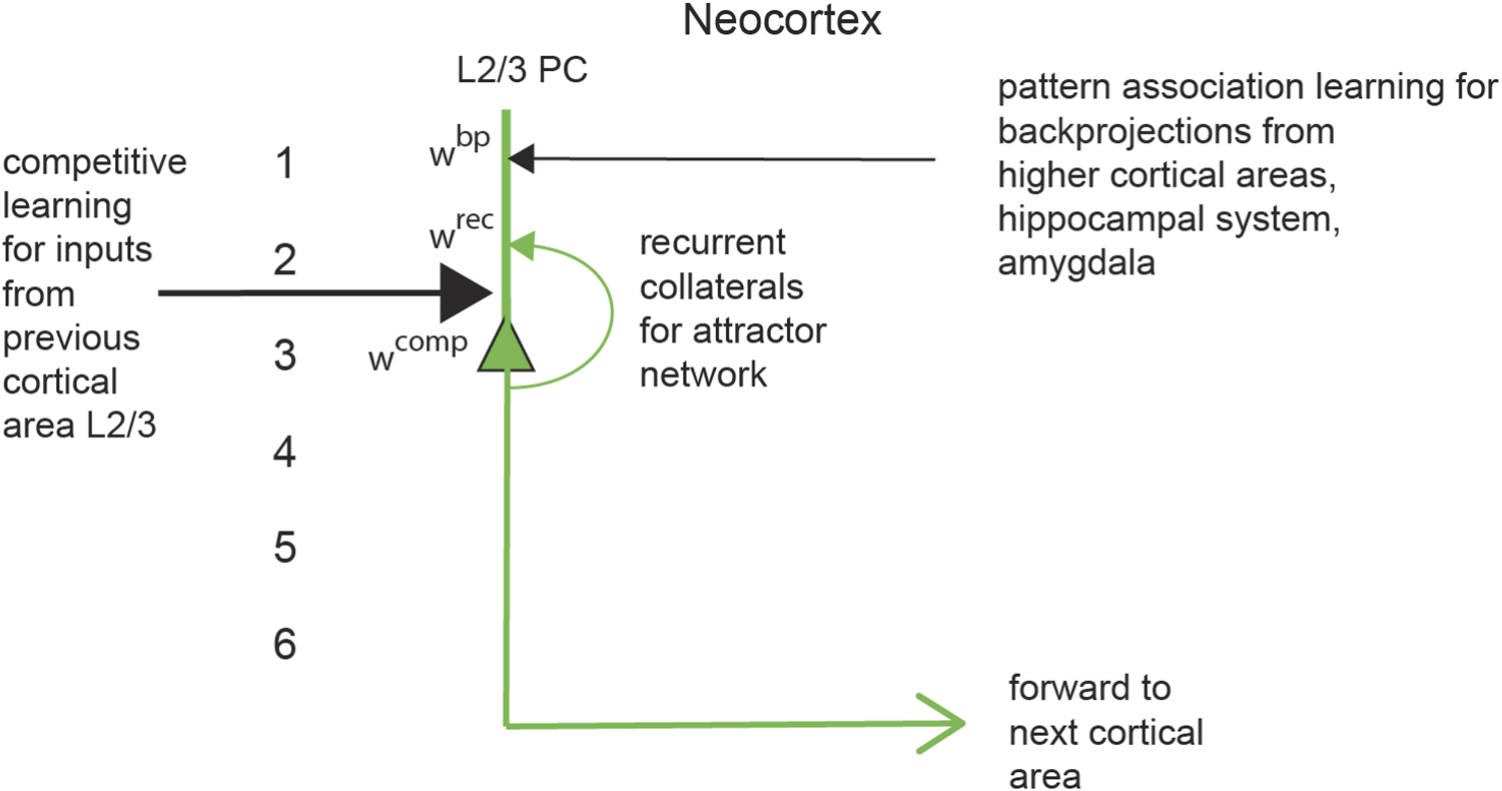
The model simulated. L2/3 PC is a layer 2/3 pyramidal cell. The thick line above the pyramidal cell body is the dendrite, receiving inputs from the previous cortical area that operate by competitive learning using the synapses *w*^comp^; from the recurrent collateral that operate as an attractor network using synapses *w*^rec^; and back-projections from higher cortical areas for memory recall and top–down attention that operate by pattern association learning using the synapses *w*^bp^. 1–6: the layers of the neocortex

First, in the architecture shown in Fig. 1, and the part considered here shown in Fig. 2, the forward inputs to a cortical module (or column) come from the previous cortical area in the hierarchy, and make synapses on the dendrites of the pyramidal cells. Some of these inputs may be relayed through granule cells in layer 4, which are hypothesised to perform expansion recoding (Rolls 2016a). (In primary sensory cortical areas, the forward inputs arrive only from the thalamus.) It is proposed that the forward inputs operate as a competitive neuronal network to build new representations, in which categorisation is performed by learning to respond to different combinations of the forward inputs (Rolls 2016a, 2021b). The properties of competitive networks are described elsewhere (von der Malsburg 1973; Rumelhart and Zipser 1985; Rolls 1989, 1992; Coultrip et al. 1992; Kaski and Kohonen 1994; Wallis and Rolls 1997; Maass 2000; Rabinovich et al. 2001; Rolls 2016a, b, 2021b, c), including online Appendices from Rolls (2016a, 2021b) at https://www.oxcns.org in which these networks are described together with code for simulations. These networks do not need to be winner takes all (with only one neuron active after the competition), and can build sparse distributed representations with a population of neurons active for any one input (Rolls 2016a, 2021b). The different neurons in the competitive network learn to respond to different combinations of inputs because the pyramidal cells are connected to inhibitory neurons (not shown in Fig. 2), which help to implement competition between the pyramidal cells by returning negative feedback to the pyramidal cells to control the level of firing to produce a sparse representation. The forward inputs to the pyramidal cells are associatively modifiable as part of this process of competitive network learning, which is described elsewhere in detail with sample tutorial code (Rolls 2016a, 2021b). A competitive network is proposed to operate for these forward inputs, because it is the main type of network with purely local learning rules that can learn new combinations of the inputs to form a categorisation neuron in an unsupervised way, that is without a teacher for each neuron (Rolls 2016a, 2021b). Categorisation refers here to the computational learning process by which correlated input patterns become pattern separated, with different (uncorrelated) input patterns allocated to different output neurons, and with similar input patterns allocated to the same output neurons (Rolls 2016a, b, 2021b). Tests of whether this categorisation has been performed successfully are whether similar input patterns are allocated to the same output neuron, and whether different (uncorrelated) input patterns are allocated to different output neurons. The implication is that the output patterns of neuronal activity (i.e. the sets of output neurons that are firing) should be less correlated, more orthogonal, than the input patterns of neuronal activity.

#### Proposal: the local recurrent collateral connections between nearby pyramidal cells implement an attractor network

Second, in this architecture illustrated in Figs. 1 and 2, the excitatory local recurrent collaterals make in the order of 10,000 synapses with other pyramidal cells within 1–3 mm, and it is proposed operate as an attractor network, with associatively modifiable synapses (Rolls 2016a, 2021b) (see “Introduction”). One property of local attractor networks is that because there are strengthened synapses between neurons that are co-active during learning, there is positive feedback between the excitatory neurons (the pyramidal cells), so that once activated during recall, the set of neurons maintains its firing when the input stimulus is removed, thus implementing a short-term memory. In the simulations to be described, the implementation of short-term memory by the recurrent collateral synapses is tested by whether the neuronal activity is maintained indefinitely after the input stimulus that initiates the recall is removed. Many different patterns each implementing a different short-term memory, can be stored in an attractor network (Hopfield 1982; Amit 1989; Treves and Rolls 1991; Rolls 2016a, 2021b). This type of attractor network implements a key computation performed by neocortical architecture, and is used for not only short-term memory and thereby for the source of top–down attention, but also for planning; and in using the property of pattern completion, is key to understanding episodic memory and semantic memory (Rolls 2016a, 2021b).

#### Proposal: the backprojections from higher cortical areas implement top–down recall and attention using pattern association

Third, in this neocortical architecture illustrated in Fig. 1, the backprojections from a higher cortical area in the hierarchy make synapses onto the apical dendrites of the pyramidal cells, and learn by pattern association learning (Rolls 2016a, 2021b), so that later when only a backprojection vector of neuronal activity is present, the pyramidal cells can be brought into activity to implement memory recall (Rolls 1989, 2021b; Treves and Rolls 1994); or can be weakly influenced to implement top–down attention, and top–down support of perceptual representations (Desimone and Duncan 1995; Deco and Rolls 2004, 2005a, b; Rolls 2008, 2016a, 2021b). The mechanism of pattern association is that the active backprojection input synapses onto the pyramidal cells in Fig. 2 become associated by associative synaptic modification onto whichever pyramidal cells are currently active. The number of such pattern associations that can be implemented is high, especially if sparse distributed representations are involved (Rolls and Treves 1990), which are present in the neocortex (Rolls and Treves 2011; Rolls 2016a, 2021b). The capacity depends on the number of inputs per neuron (Rolls and Treves 1990), and because in a memory system it is necessary to be able to recall as many memories as can be stored, this provides the only quantitative theory of why there are approximately as many backprojection inputs to each neocortical pyramidal cell as there are forward inputs (Treves and Rolls 1994; Rolls 2016a, 2018, 2021b). The operation of top–down recall can be tested by presenting the vectors of neuronal activity used during the original learning as recall cues later, to test whether the correct pyramidal cells are activated that were originally activated when the corresponding forward input was presented during learning.

#### Proposal: these three computations can be combined using neocortical pyramidal cells with their three types of connection

Each of these neuronal network architectures, competitive learning, attractor networks, and pattern association learning, are normally investigated separately, and programs to illustrate their operation separately are available (Rolls 2016a, 2021b) (https://www.oxcns.org). A key part of the theory proposed here of the computational operation of neocortical pyramidal cells is that these three neuronal architectures can operate together, on the same single set of neurons, in this case pyramidal cells. One particular issue that is investigated in the simulations is whether the system illustrated in Fig. 2 can self-organise, with just sets of forward inputs being presented, and the corresponding backprojection inputs presented at the same time. Self-organisation of the neocortex seems to be a fundamental neocortical property, in that there is no teacher for each output neuron, and no system that could easily implement error backpropagation (Rolls 2016a, 2021b, c).

Another particular issue that arises is whether all three types of computation can coexist on the same neurons, given that normalisation of the length of the synaptic weight vector of each neuron is useful in competitive networks to ensure that the neurons compete equally (Rolls 2016a, 2021b). This is an issue that has not been previously investigated. To address this issue, and to test the whole theory, and to make very clear and explicit the theory of the operation of neocortical pyramidal cells described here, simulations of the architecture illustrated in Fig. 2 are described here. The implementation of the simulations, and then the results obtained, are described next. The simulations investigate also the relative strengths of the different inputs that enable the three types of learning to be successfully implemented, which is a key part of the quantitative understanding of how neocortical pyramidal cells may operate to implement these three different types of computation.

### The computational model simulated

The model simulated had the architecture shown in Fig. 2, and was a rate model. The same associative learning rule was used at every synapse, namely

1 $${\updelta }w_{ij} = {\upalpha }y_{i} x_{j} ,$$

where ${\updelta }w_{ij}$ is the change of the synaptic weight of the *j*’th synapse on the *i*’th neuron, ${\upalpha }$ is the learning rate, $y_{i}$ is the firing rate of the *i*’th neuron, and $x_{j}$ is the presynaptic firing rate of the *j*’th input.

In more detail, the synapses for the input from the previous cortical area were trained with the rule

2 $$\delta w_{ik}^{{{\text{comp}}}} = \alpha^{{{\text{comp}}}} y_{i} x_{k} ,$$

where $\delta w_{ik}^{{{\text{comp}}}}$ is the change of the synaptic weight of the *k*’th synapse for the input from the previous cortical area on the *i*’th neuron, $\alpha^{{{\text{comp}}}}$ is the learning rate, $y_{i}$ is the firing rate of the *i*’th neuron, and $x_{k}$ is the presynaptic firing rate of the *k*’th input from the previous cortical area.

The recurrent collateral synapses for the attractor system were trained with the rule

3 $$\delta w_{il}^{{{\text{rec}}}} = \alpha^{{{\text{rec}}}} y_{i} x_{l} ,$$

where $\delta w_{il}^{{{\text{rec}}}}$ is the change of the synaptic weight of the l’th synapse for the recurrent collateral inputs from the set of pyramidal cells in the layer on the *i*’th neuron, $\alpha^{{{\text{rec}}}}$ is the learning rate, $y_{i}$ is the firing rate of the *i*’th neuron, and $x_{l}$ is the presynaptic firing rate of the input from the *l*’th pyramidal cell in the same layer.

The backprojection synapses were trained with the rule

4 $$\delta w_{im}^{{{\text{bp}}}} = \alpha^{{{\text{bp}}}} y_{i} x_{m} ,$$

where $\delta w_{im}^{{{\text{bp}}}}$ is the change of the synaptic weight of the *m*’th backprojection synapse on the *i*’th neuron, ${\upalpha }^{{{\text{bp}}}}$ is the learning rate, $y_{i}$ is the firing rate of the *i*’th neuron, and $x_{m}$ is the presynaptic firing rate of the *m*’th backprojection input from the next cortical area or the amygdala or the hippocampal system.

During training, after the synapses had been associatively modified as just described, the length of the synaptic weight vector of each neuron was normalised (Rumelhart and Zipser 1985). This is a useful step in competitive network learning, as it has the effect of ensuring that each neuron can compete equally. The normalisation has the effect of decreasing the synaptic strength of inactive synapses to activated neurons, which is a type of heterosynaptic long-term depression that is biologically plausible (Rolls 2016a, 2021b). Another formulation that achieves the same has been described (Oja 1982).

The activations of the *i*’th neuron $h_{i}$ were calculated as a synaptically weighted sum of the input firing rates multiplied by the synaptic weights:

5 $$h_{i} = \sum\nolimits_{j} {x_{j} w_{ij} }$$

In more detail, the separate component inputs to a pyramidal cell *i* add as follows:

6 $$h_{i} = \sum\nolimits_{k} {x_{k} w_{ik}^{{{\text{comp}}}} } + \sum\nolimits_{l} {x_{l} w_{il}^{{{\text{rec}}}} } + \sum\nolimits_{m} {x_{m} w_{im}^{{{\text{bp}}}} }$$

There were separate fixed scale factors for the three types of synapse onto a neuron, forward $w_{ik}^{{{\text{comp}}}}$, recurrent $w_{il}^{{{\text{rec}}}}$, and backprojection $w_{im}^{{{\text{bp}}}}$, with the recurrent and backprojection synapses set to a low value (0.1 as the default, but explored as described in the “Results”) as they should leave the forward inputs to dominate the activations during learning, but should support short-term memory and recall, respectively, when there are no forward inputs. These scale factors might be set in the cortex by the size of the type of synapse, whether it is close to or far from the cell body, etc. Each type of input had nSyn synapses, resulting in 3 * nSyn inputs to each neuron. nSyn was set by default to the same value as N, that is, to 100. To initialise the network, all synapses were set to uniform random values in the range 0–1, and then the length of the synaptic weight vector on each output neuron was normalised (i.e. set to a length of 1). This and the positive training patterns ensured that all synaptic weights were greater than or equal to zero.

Similarly, there were separate fixed scale factors for the learning rates $\alpha^{{{\text{comp}}}} ,$ etc. for the three types of input to a neuron, forward, recurrent, and backprojection (see Eqs. 2–4), to allow the synaptic strengths to be comparable for these three types of input. A threshold binary activation function was used to achieve a fixed sparseness of the firing of the population of *N* neurons. (Sparseness for binary neurons is the proportion of neurons with high firing rates (i.e. 1 in a system with rates that are 0 or 1) (Treves and Rolls 1991; Rolls 2021b).) Setting the sparseness in this way simulated the effect of inhibitory negative feedback neurons to the population of pyramidal cells, as the operation of inhibitory interneurons in competitive networks (Coultrip et al. 1992) was not a property under investigation here. The competition between the pyramidal cells is implemented in this way, in that only the pyramidal cells with the highest activations are left firing after the competitive interaction implemented by setting the sparseness of the output representation has been performed.

**Fig. 3:**
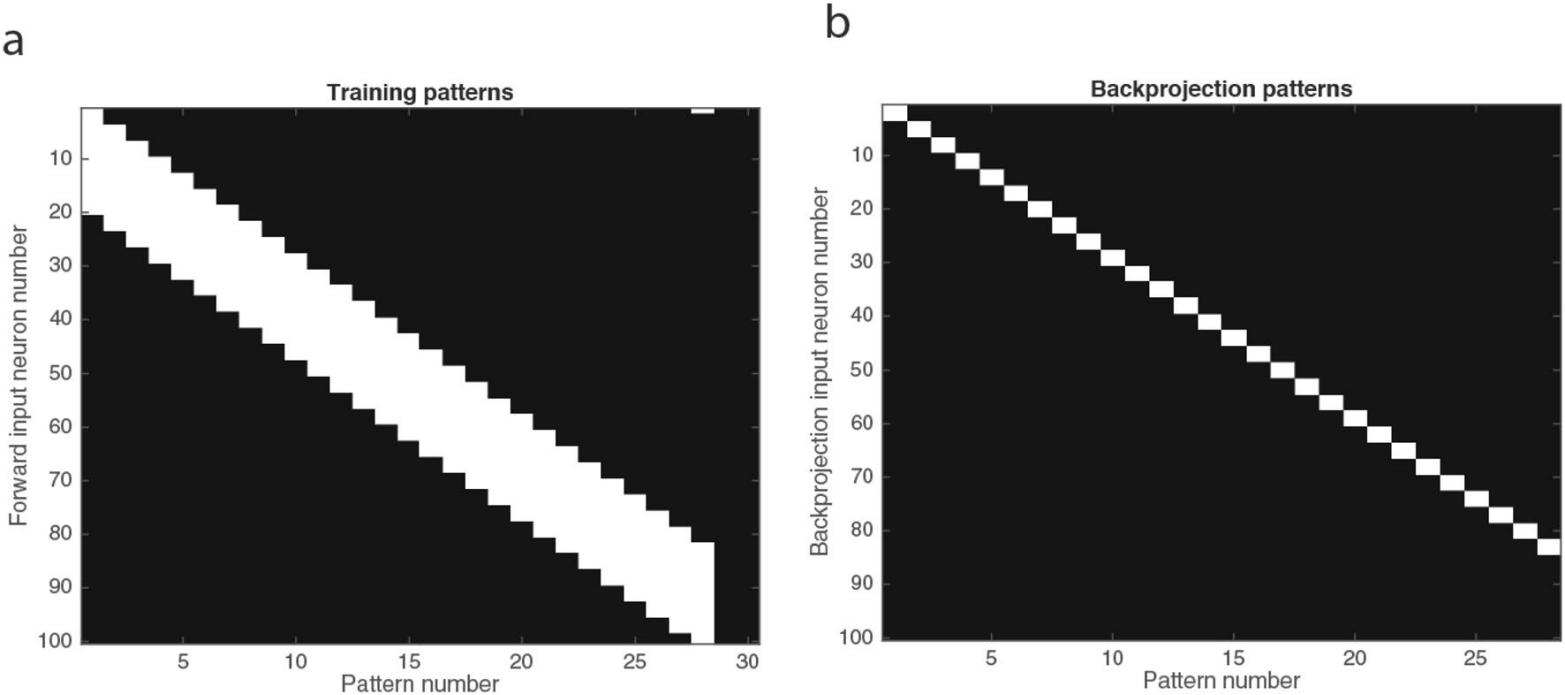
**a** The 28 training patterns used for the forward inputs. Each pattern involved a high rate of 20 of the 100 input neurons to the competitive network. Each input pattern overlapped by 17 locations with the closest training pattern. A high rate is indicated by white, and zero by black. The 100 inputs were used for the training of the competitive network forward input to the pyramidal cells. Each pyramidal cell had 100 synapses devoted to these forward inputs, so this was a fully connected competitive network. The mean correlation between these training patterns was 0.31. **b** The 28 training patterns used for the backprojection inputs used to recall the categorised representation produced by the network in its output neurons. Each backprojection pattern was 3 neurons long out of the 100 backprojection inputs and was applied to the 100 backprojection synapses on each pyramidal cell. Each backprojection pattern overlapped by 0 locations with the closest backprojection pattern

The forward input vectors were 28 binary overlapping patterns as illustrated in Fig. 3a, and were chosen to test whether the network could pattern separate these into orthogonal categories, with similar patterns allocated to the same category. The mean correlation between these training patterns was 0.31. Other types of input pattern, including random patterns, can be explored with the software provided. The top–down backprojection patterns were 28 orthogonal vectors each with a length of 3 as illustrated in Fig. 3b. The code available in the Supplementary Material as CompetitiveAttractorBPNetDemo.m allows all of these parameters to be explored. The program also provides for the use of random binary inputs for the forward input vectors. The purpose of what is set out in this paper is to propose the concepts of operation of this system, with full-scale simulations of a cortical area left for future work.

The network was trained for five epochs. In each epoch, each of the 28 forward input patterns with the corresponding backprojection patterns was selected in random permuted sequence, the firing rates were calculated based on the relevant presynaptic rates and synaptic weights, and the synapses were updated as shown in Eqs. 2–4.

Then, in testing, the network was presented with the forward inputs, to test whether the categorisation of the inputs had been achieved correctly by the competitive network. Correct operation for the set of overlapping input stimuli was that the outputs should be categorised such that similar patterns were placed into the same category, and that the outputs should be less correlated than the inputs. Each forward input was presented once to test the categorisation, the forward input was then switched off, and the network was allowed to run for 10 iterations (or any number) to test whether the recurrent collaterals operated correctly as an attractor network to maintain the firing rates in a short-term memory with stable retrieval, i.e. in a stable fixed point.

To test whether the top–down inputs had learned correctly by pattern association learning, each of the 28 backprojection inputs was presented serially in turn with no forward input, to investigate whether the categorised outputs appropriate for each of the 28 forward inputs were correctly recalled by the backprojection inputs.

The predictions that were tested in the simulations were as follows. First, in the architecture shown in Fig. 2, it is predicted that the network can be trained with the forward inputs operating as a competitive network to categorise the input patterns, even when the recurrent collaterals synapses are operating and learning, and when the backprojection inputs are being applied. Categorisation is measured by whether the patterns of the output rate vector, i.e. the set of active output neurons for a given forward input, are more orthogonal (i.e. less correlated) than the forward input rate patterns, and whether similar input patterns are allocated to the same output neurons. For this to occur, it is expected that the (fixed) scale factors for the forward inputs will need to be greater than for the recurrent collateral and backprojection synapses both of which operate during learning. Second, it is predicted that the recurrent collateral connections will form an attractor network that can maintain the firing of the pyramidal cells when the forward and backprojection inputs are removed. Third, it is predicted that the backprojection inputs present during the learning will later be able to recall the set of rates of the output neurons that were self-organised during the competitive learning of the forward inputs when the backprojection inputs were also present. Fourth, it is predicted that a set of (fixed) scale factors for the three types of synaptic input to a neuron shown in Fig. 2 and specified in Eq. 6 can be found that will allow correct learning of the output representations produced by the forward inputs and that will allow all three types of synapse to perform the functions described. Fifth, it is predicted that the synaptic weight normalisation used for the competitive learning can be applied to the whole set of synaptic inputs to each neuron without interfering with the attractor and recall functions for the recurrent and backprojection synapses.

The full details of the implementation of the network, and the results that are obtained with it, are evident in the Matlab program CompetitiveAttractorBPNetDemo.m that accompanies this paper.

## Results

### Results of training

**Fig. 4:**
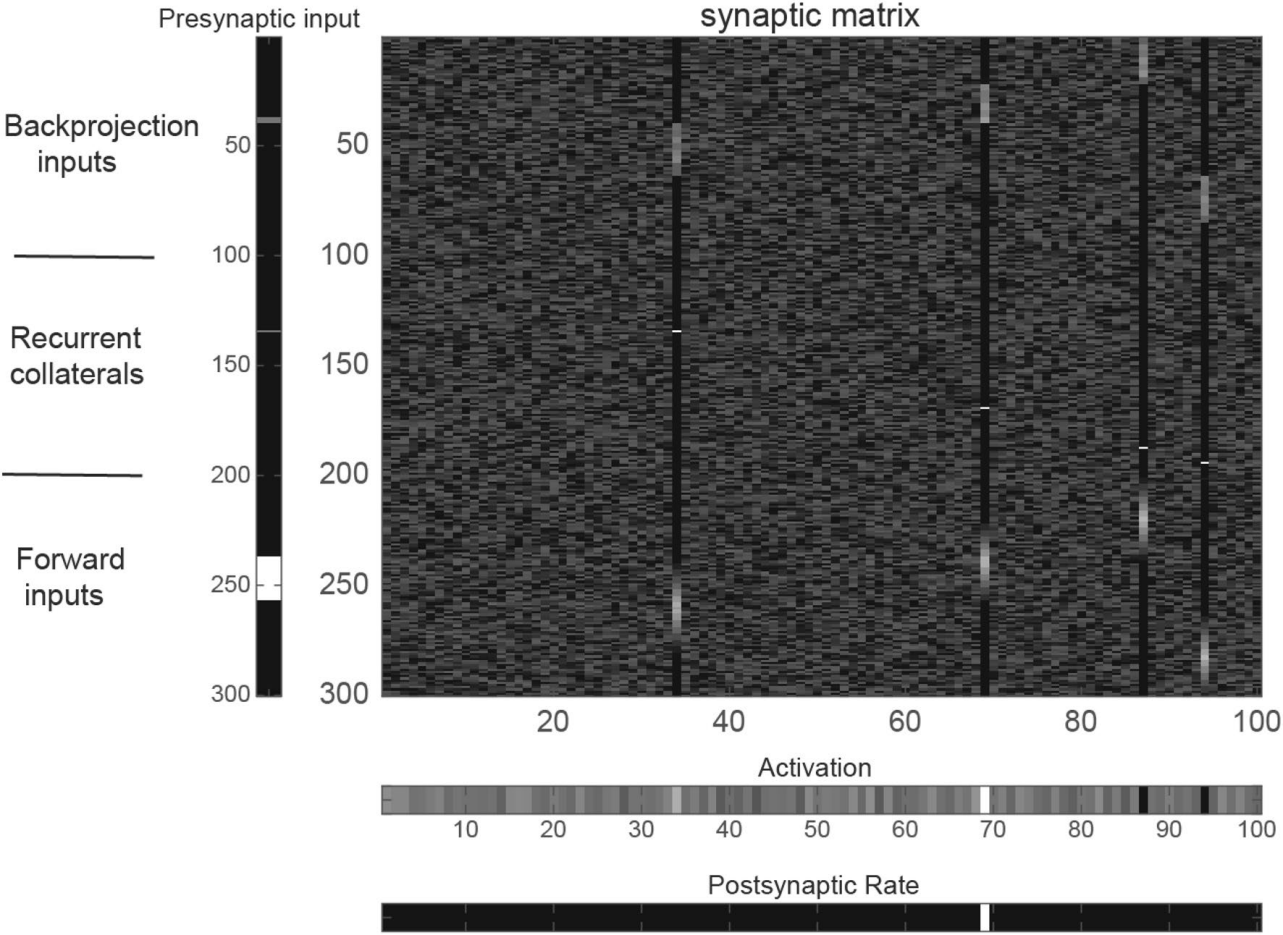
The synaptic matrix in the neocortical network model can self-organise to categorise the input stimuli into four categories; the recurrent collaterals learn to implement an attractor network for short-term memory; and the backprojection synapses learn to implement top–down recall. The activations and rates of the *N* = 100 neurons are shown in the bottom two vectors. The presynaptic input vector of rates is shown on the left, with the top nSYN = 100 rows the backprojection input; the middle nSyn = 100 rows the recurrent collateral input vector to the neurons; and the bottom nSYN = 100 rows the forward input vector. High firing rates of the presynaptic neurons are shown in white. In the synaptic matrix with 300 rows for the synapses on each of the *N* = 100 dendrites shown as the columns, a high synaptic weight is indicated by white, and a zero synaptic weight by black. The activations of the neurons produced by the presynaptic input are shown in a row near the bottom of the figure, with black an activation of 0. The firing rates of the neurons produced by the binary threshold activation function are shown in the row at the bottom of the figure, with the numbers identifying the neuron number, and black specifying a firing rate of 0, and white of 1. The sparseness of the output rate representation is *N*/100. In the synaptic matrix, most of the synapses reflect the random initial values, with the minimal value in the matrix 0 and shown as black. For the four neurons that have learned, it is possible to see near the top of the dendrite (the column of synaptic weights) the strengthened synapses that implement the recall produced by the backprojection inputs. Near the middle of each dendrite can be seen the synaptic weights that implement the attractor network. In this simple simulation, there is just one synapse used for this for each neuron, because there is only ever one output neuron firing during learning, as the sparseness has been set for didactic purposes to produce a single winning neuron. This can be altered in the program by altering the sparseness of the output. Near the bottom of each dendrite are the synapses that have modified in the competitive part of the network to make a neuron respond to any one of a set of similar (overlapping) forward input patterns

The 28 training patterns are shown in Fig. 3a, and the 28 backprojection patterns used for recall are shown in Fig. 3b. The synaptic matrix after training for five epochs (each epoch consisting of every input pattern and its associated backprojection input being presented once in random permuted sequence) is illustrated in Fig. 4. The matrix of synaptic weights was initially random, but it can be seen that some neurons have learned with high synaptic weights associated with some inputs, and low synaptic weights elsewhere (as shown by black for the rest of the dendritic column which indicates a synaptic weight of 0). The other neurons with random synaptic weights remain unallocated and available for use by further sets of input stimuli. This is an important property of competitive networks, that not all the neurons are affected by the training for a given set of stimuli and sparseness of the output, and remain available for other stimuli in future (Rolls 2016a, 2021b).

### Learning of new categories by the competitive system

**Fig. 5:**
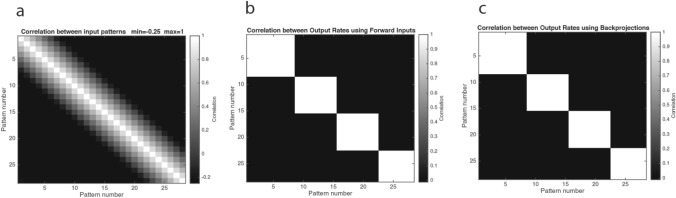
**a** The correlation matrix between the input patterns shown in Fig. 3a. The mean correlation between these training patterns was 0.31. **b** The correlation matrix between the output neuronal rate patterns from the population of 100 output neurons when tested with the 28 forward inputs shows that the 28 input patterns were categorised into 4 categories, with no overlap between the categories. The mean correlation between these output neuronal rate patterns of the neuronal population was 0.01. Moreover, adjacent input patterns are placed into the same category. **c** The correlation matrix between the output neuronal rate patterns from the population of 100 output neurons when tested with the top–down backprojection inputs shows that the 4 categories of output produced by the 28 forward input patterns were recalled perfectly by the backprojection inputs. The mean correlation between these output rate patterns of the neuronal population was 0.01

The competitive network operated as expected even though this was a combined architecture, and learned in this case 4 categories of output rate vectors for the 28 overlapping input patterns. Figure 5b shows that the output patterns of neuronal activity were orthogonal, and moreover, that similar input patterns were placed into the same output category. (An output pattern of neuronal activity here refers to the set of output neurons that were active with high rates for a given input.) The correlations between the input patterns in contrast were high, as shown in Fig. 5a, with a mean correlation of 0.31. The operation was thus as expected of a competitive network, with correlated input patterns categorised into orthogonal categories, with each category containing similar patterns to each other. Part of the importance of this is that the network operated as a competitive network to implement categorisation of the forward input patterns even though the same neurons in the networks were simultaneously learning to operate as an attractor network, and were learning to operate as a network that could recall its output using the backprojection inputs. Further details on the quantitative operation of competitive inputs with random input vectors to further illustrate the operation of competitive networks are provided elsewhere (Rolls and Treves 1998; Rolls 2021b), and the program CompetitiveAttractorBPNetDemo.m allows random input patterns to be chosen for simulation.

### Short-term memory

The attractor network functionality operated perfectly even though this was a combined architecture, with the output firing produced by the forward input continuing indefinitely without change when the forward input was removed. This is illustrated in the program CompetitiveAttractorBPNetDemo.m, and is not further illustrated, as the firing remains the same as that illustrated in Fig. 5b when the forward input is removed. Part of the importance of this is that the network could learn how to set up a recurrent collateral network for attractor dynamics at the same time as it was learning to categorise the forward inputs using competitive learning, and was learning the correct synaptic connections for the backprojection recall to operate. That is, the attractor synapses and the backprojection synapses, both active and being modified during the competitive learning using the forward inputs, did not disrupt the competitive learning using the forward inputs.

### Recall

Figure 5c shows that the output produced by each of the 28 recall vectors was the exact categorised output rate that was produced by the forward input after learning. Thus, the recall implemented by the backprojections operated perfectly even though this was a combined architecture. Part of the importance of this is that the network could learn how to set up the backprojection synapses at the same time as it was learning to categorise the forward inputs using competitive learning. That is, the backprojection synapses, active and being modified during the competitive learning using the forward inputs, did not disrupt the competitive learning using the forward inputs.

### Top–down attention

Top–down attention is implemented in this system using the backprojection inputs. Top–down attention to one of the stimuli would be produced by a weak backprojection signal, which produced weak activation of some of the neurons through the backprojection synapses trained as described here. If the forward inputs that are applied simultaneously are also weak, then the input stimulus that wins the competition is influenced by the top–down, back-projected, recall signal. The exact regimes in which these interactions between the top–down and bottom up signals produce top–down attention have been analysed elsewhere (Deco and Rolls 2005b; Turova and Rolls 2019). The architecture in Fig. 2 shows how this would be implemented in the cortical pyramidal cell system described here.

### Operation of the neocortical network

What was of considerable interest was the parameters with which these results were obtained, and that the network operated well when all three classes of input were included in the same synaptic weight normalisation applied to each neuron. The prior hypotheses were that the forward input would have to be relatively strong, with a relatively high learning rate, to support the competitive learning, without dominance by the other inputs to the neurons, the recurrent and back-projection inputs. It was also hypothesised that the recurrent collaterals should not have too high an input to the neurons, so that they did not influence the competitive learning, but would also, when there was no forward input or top–down recall input, be able to maintain the firing of the output neurons in the attractor network indefinitely. It was also hypothesised that the top–down backprojection input should have only a relatively weak effect on the neurons, so that it would not influence the competitive learning, but would be sufficient in the absence of a forward input to initiate recall of the correct category of output neuron firing.

The results of running the simulation showed that the system operated well when the (fixed) scale factor of the recurrent collateral inputs relative to the forward inputs was in the region of 0.1 (with considerable tolerance within a range of 0.02–0.2); and when the (fixed) scale factor of the backprojection inputs relative to the forward inputs was 0.1 (with considerable tolerance within the range 0.1–0.15). These scale factors for the different classes of input to a neuron might be set biologically by processes such as the fact that the backprojection inputs are received on the apical dendrites in layer 1, and will tend to be shunted by any forward inputs lower down the dendrite and closer to the cell body.

The fixed parameter learning rate $\alpha^{{{\text{comp}}}}$ for the competitive network synapses was in the order of 0.1 (and depends on the number of categories being formed, with too high a value tending to overwrite previous learning by placing too much emphasis on the current forward input pattern). The learning rate for the backprojection synapses was also in the order of 0.1 (which satisfied the aim of producing some high value backprojection synapses). The learning rate for the recurrent collateral synapses was in the order of 0.03, with again the criterion that it must be sufficiently strong to produce some high value synaptic weights.

The program runs with a new random seed every time it is run. There is, therefore, some difference from run to run, due to the different random initialisation of the synaptic weights. Usually, four categories are formed from the 28 forward input patterns, but sometimes five, with the standard set of parameters. The actual neurons that respond to each category of forward input stimulus vary from run to run. These results show that the findings described are computationally robust. Moreover, the range of parameters within which similar generic results was found was as described quite wide, providing further evidence on the robustness of the findings and network described here.

## Discussion

The important conclusion of this research is that it is possible to combine in the same neurons in a model of neocortical computation: (1) categorisation using competitive learning; with (2) attractor network dynamics to maintain the firing when the forward input is removed; and with (3) pattern association learning of backprojection inputs to implement memory recall (and the related effects such as top–down attention (Rolls 2016a, 2021b)). This is extremely interesting as a conceptual computational model of the cerebral neocortex, because all of these operations can be learned simultaneously using the same set of neurons. This is thus a model of how just one excitatory neuron type in the neocortex, the pyramidal cells, can perform these three different types of computation that are key components of neocortical function. This produces an important step forward in our understanding of how the neocortex may operate to perform its key computational functions.

The research also showed that the system operates well when all the synaptic weights are initialised to random values. Such random synaptic weights or diluted connectivity are important for competitive learning, by breaking the symmetry between neurons (Rolls 2016a, b, 2021b). But normally for attractor networks and pattern association networks the synaptic matrix starts with zero initial weights. It is shown here that the system still learns correctly when for generality these synapses do not start at zero, and are subject to synaptic weight normalisation.

The research also shows that the synaptic weight normalisation that is typically used in competitive networks (Rolls 2016a, 2021b) does not present problems in this combined architecture when this is applied to the whole length of the dendrites, including the parts utilised by the recurrent collaterals and backprojection pattern association synapses. This is a useful result shown by this research, for it indicates that specialisation of different parts of the dendrite receiving different classes of input is not required. This makes the current approach to understanding how a neocortical module computes biologically plausible.

There is considerable research that each of the computational processes described here is important for understanding the operation of the cerebral cortex.

For example, competitive learning is the key computation that when combined with slow learning over transforms of objects enables transform-invariant visual object recognition to occur in a model of the ventral visual system, VisNet (Rolls 2012, 2021b; c).

Pattern association learning between backprojections from the hippocampal system to the neocortex (Rolls 1989) provides the only quantitative and analytic theory and model (including capacity, the number of memories that can be recalled) of how information is recalled from the hippocampus through the multistage pathway via the entorhinal cortex, perirhinal/parahippocampal cortex, to neocortical pyramidal cells (Treves and Rolls 1994; Rolls 2018, 2021b).

Top–down influences of the type implemented by backprojections are fundamental for biologically plausible models of top–down attention (Deco and Rolls 2004, 2005a) by biased competition (Desimone and Duncan 1995) and biased activation (Rolls 2013).

Cortical attractor networks implemented by recurrent collateral synapses between the principal neuron, pyramidal cells, provide a model for short-term memory (Wang 1999; Rolls and Deco 2015b), long-term memory in the hippocampus and neocortex (Rolls 2018, 2021b), and for decision-making (Wang 2002; Rolls and Deco 2010; Rolls et al. 2010; Deco et al. 2013; Rolls 2021b).

Given that there is one principal excitatory neuron type in the neocortex, the pyramidal cell (Rolls 2016a), the research described here is conceptually an important advance, for it shows that all these types of computation can be performed with one type of single neuron with the connections and properties that are prototypical of pyramidal cells of the cerebral neocortex (Rolls 2016a, 2021b). The key aim of this paper has been to propose this concept: that all three types of computation can be performed with pyramidal cells, as found for example in layers 2 and 3 of the neocortex. This is the first time as far as I know that these three computations have been proposed for a single type of neuron, neocortical pyramidal cells, for this is consistent with cortical anatomy. It is also the first time I know that these three computations, of competitive learning, attractor networks, and backprojections for recall by pattern association have been shown to be compatible with each other on a single type of neocortical neuron, the pyramidal cell, to accomplish some of the key types of different computation performed by the cerebral neocortex.

An important part of the computational proposal made here is that when new learning is taking place in a population of cortical neurons, the forward inputs being received from the previous cortical area and that may reflect perceptual or reward-related inputs will dominate the firing of the pyramidal cells, with the relatively weaker recurrent collateral and backprojection inputs not contributing much to the firing of the pyramidal cells, though the recurrent and backprojections synapses can nevertheless learn as they have the appropriate presynaptic and postsynaptic signals. Indeed, for top–down biased competition to operate well, the top–down inputs received by the cortico–cortical backprojection inputs need to be much weaker than the bottom–up forward inputs (Deco and Rolls 2005b; Rolls 2016a; Turova and Rolls 2019). When bottom–up forward inputs are removed, the recurrent collateral synapses are then sufficient to maintain the network in an attractor, to implement short term memory, as there is no domination by bottom–up inputs. When bottom–up inputs are not present to dominate the pyramidal cell neuronal activity, then the top–down backprojection inputs are sufficiently strong to initiate recall, as shown here.

There are some interesting potential implications for understanding cortical design of the concepts developed here. One is that a cortical area does not need to have different types of neuron specialised for different functions such as learning new representations, implementing short-term memory, and implementing recall using backprojections from for example the hippocampal system (Rolls 2018, 2021b). Instead, all of the computations can be combined onto one type of neocortical cell, the cortical pyramidal cell, which is the main excitatory neuron type in the neocortex. This leads to economy of genetic specification of different excitatory neuron types and of all the connections required between the different specialised populations. And after all of that computation if it was performed by separate computational units, the different types of computational unit might need to project to another neuron type that could then respond to all these types of input and then transmit the output up the hierarchy, and back down the hierarchy. Instead, it is, it appears, far more efficient to have one main excitatory neuron type, the pyramidal cell, in neocortical areas. Then all the three types of computation described here communicate with each other simply, because all are implemented in the same cells, which then have a single output that can be projected forward up the hierarchy, or for deep pyramidal cells backwards down the hierarchy. And further, it may be advantageous to have a single post-synaptic term for learning inside each neuron, for then the signal can be applied to all the different types of input synapse without further communication required than current spread within the neuron. Moreover, it can be advantageous to have these different computations performed by the same neuron, for the different computations may be important for each other, with for example slow learning which can benefit from a short-term memory trace implemented by the recurrent collaterals to learn invariant representations of stimuli and other properties that can benefit from some continuity in time to benefit from the statistics of the natural environment (Rolls 2021b, c). The great economy in the design of the neocortex can of course be varied quantitatively from cortical area to cortical area, with for example some brain areas specialised for short-term memory (the prefrontal cortex) or semantic memory (the anterior temporal lobe), and having accordingly larger numbers of recurrent collateral synapses on each neuron, and correspondingly larger dendritic trees (Elston 2007; Rolls 2016a, 2021b).

In future research, it will be of interest to further investigate the operation of the system described here, for example by further parameter exploration, and tests of a scaled up version. One point of interest, and a possible limitation, is that for competitive networks, it is useful to normalise the length of the synaptic weight vector on the dendrite of each neuron (Willshaw and von der Malsburg 1976; Hertz et al. 1991; Rolls 2021b), to help the neurons to compete equally. However, a possible physiological implementation has been proposed (Oja 1982), and simpler constraints such as that the total strength of the synaptic inputs to a neuron may be limited by the number of synapses and their strengths could be further explored, as could the utility for this of heterosynaptic long-term depression (Rolls 2021b). It was interesting to show in the research described here that if synaptic weight normalisation is used, it is not incompatible with the operation of the recurrent collateral attractor property and backprojection recall property described here. In future neurophysiological research to test the theory described here, it will be of interest to investigate not only the types of heterosynaptic long-term depression that may be present, but also if the relative scale factors for the forward inputs from the previous cortical area, for the local recurrent collaterals, and for the backprojection synaptic inputs, for neocortical pyramidal cells, are as predicted here. In addition, although there is considerable neurophysiological research to show that cortical neurons can usefully categorise new inputs during learning (Rolls et al. 1989), can maintain activity after a stimulus is removed (Rolls and Tovee 1994; Goldman-Rakic 1996; Rolls et al. 1999; Rolls 2003; Miller 2013), and can recall cortical activity when a recall cue is provided, further investigations relating to the neuronal implementation proposed here would be very interesting. In future research, it will also be of interest to examine whether similar principles apply to the pyramidal cells in layer 5. An interesting difference is that the layers 2 and 3 pyramidal cells tend to project forward to the next cortical area, typically up through a hierarchy; whereas the layer 5 pyramidal cells tend to provide the backprojections to the previous cortical area in the hierarchy, and to subcortical regions such as the striatum (Fig. 1) (Rolls 2016a, 2021b). This is likely to be important in understanding the different computational roles of the superficial and deep pyramidal cells of the neocortex, which are incompletely understood (Markov et al. 2013; Rolls 2016a, 2021b; Rolls and Mills 2017). Overall, the principles of operation of neocortical pyramidal cells described here which include categorisation in cortical hierarchies, local short-term and long-term memory implemented by the recurrent collaterals, and memory recall and top–down attention implemented by the backprojections provide a foundation for understanding many aspects of cortical function (Rolls 2016a, 2021b, c). Understanding the operation of neocortical circuitry is also of clinical relevance, with reduced activity of cortical neurons leading it is proposed to decreased stability of cortical networks producing some of the symptoms of schizophrenia (Rolls 2021a) and ageing (Rolls and Deco 2015b); and reduced forward effective connectivity relative to backward effective connectivity implicated in the greater dominance of self-generated internal thoughts relative to inputs from the world in schizophrenia (Rolls et al. 2020).

## Supplementary Information

Below is the link to the electronic supplementary material.

Supplementary file1 (ZIP 6 kb)

## Data Availability

The Matlab software developed for this research is available in RollsMatlabSoftware.zip at https://www.oxcns.org/publications.html as the Supplementary Material for this paper.
